# 4‐Methylumbelliferone (4‐MU) Improves Learning and Memory After Cerebral Ischemia/Reperfusion Injury in Rats

**DOI:** 10.1155/bn/5900565

**Published:** 2025-12-05

**Authors:** Shaghayegh Tamouk, Hamzeh Mirshekari Jahangiri, Elham Kashafi Jahromi, Michael R. Hamblin, Nahid Aboutaleb, Fatemeh Ramezani

**Affiliations:** ^1^ Physiology Research Center, Iran University of Medical Sciences, Tehran, Iran, iums.ac.ir; ^2^ Department of Physiology, School of Medicine, Iran University of Medical Sciences, Tehran, Iran, iums.ac.ir; ^3^ Laser Research Centre, Faculty of Health Science, University of Johannesburg, Doornfontein, South Africa, uj.ac.za

**Keywords:** 4-methylumbelliferone, hyaluronan synthases, ischemic stroke, learning and memory impairments, MCAO, neuronal death

## Abstract

**Background:**

Stroke is the sixth leading cause of death and lifelong disability for millions of people in the United States. Cerebral ischemia leads to oxidative stress, excitotoxicity, inflammation, and apoptosis; additionally, impairment in memory and learning occurs in the majority of subjects with ischemic stroke. The lack of definitive treatment has sparked extensive research into novel therapeutic strategies, including the use of 4‐methylumbelliferone (4‐MU), a coumarin derivative with potential neuroprotective properties. The present study examines the impact of 4‐MU on reducing cerebral ischemia–reperfusion (I/R) injury and learning and memory impairments in male Wistar rats.

**Methods:**

Animals were exposed to middle cerebral artery occlusion (MCAO) and treated with a single dose of 4‐MU (25 mg/kg) dissolved in 0.9% DMSO. An automated shuttle box and Morris water maze (MWM) tests were employed to evaluate learning and memory impairments. Western blot assay, TTC staining, and Nissl staining were used to measure protein expression, infarct volume, and cell death, respectively.

**Results:**

Treatment with 4‐MU reduced infarct volume and improved learning and memory impairments by downregulating HAS1 and HAS2. 4‐MU modulated the release of proinflammatory cytokines including TNF‐*α* and IL‐1*β*, as well as anti‐inflammatory markers like IL‐10, and reduced oxidative stress markers in the brain.

**Conclusion:**

The neuroprotective effects of 4‐MU against cerebral I/R injury can be attributed to the downregulation of HAS1 and HAS2.

## 1. Introduction

One of the main outcomes following ischemic stroke is impairment in memory and learning, which strongly influences patients′ quality of life [1]. Previous investigations have shown that cerebral ischemia for a short time (less than 10 min) contributes to the initiation of cell death in the hippocampus and causes deficits in learning and memory functions [2, 3].

A large number of individuals who survive the acute phase of ischemic stroke encounter adverse effects related to deficits in memory and learning because available treatments are not completely effective [4]. According to clinical studies, immediate or delayed dysfunctions in memory and learning occur in 25%–30% of poststroke survivors, which heavily influences rehabilitation programs for activities of daily living [5, 6].

Poststroke cognitive impairment affects 25%–30% of stroke survivors and represents a major barrier to functional recovery and quality of life. The hippocampus, particularly the CA1 region, is highly vulnerable to ischemic injury, with even brief episodes of cerebral ischemia capable of triggering neuronal death and persistent memory deficits. Despite the clinical significance of poststroke cognitive impairment, effective therapeutic interventions remain limited, underscoring the critical need for novel neuroprotective strategies targeting cognitive preservation following cerebral ischemia.

Hyaluronan (HA) is a key ingredient of the brain extracellular matrix and plays a crucial role in processes like angiogenesis, cellular proliferation, differentiation, and migration. Increased synthesis of total HA and low molecular mass 3–10 disaccharides of HA (o‐HA) in the serum of patients with ischemic stroke (1, 3, 7, and 14 days) and postmortem tissues has been reported in prior investigations, mainly attributed to overexpression of HA synthases (HAS1 and HAS2) and hyaluronidases in inflammatory cells in peri‐infarcted areas of the cerebral tissue [7]. Elevated expression of hyaluronidase‐1 and HAS2 in astrocytes following ischemic stroke aggravates infarct volume, and suppressing hyaluronidase activity early after stroke can contribute to functional recovery [8].

4‐Methylumbelliferone (4‐MU), an inhibitor of hyaluronic acid synthesis, has been found to have a protective impact against ischemia/reperfusion injury and other disorders by inhibition of oxidative stress and inflammation [8–12]. The protective impact of 4‐MU is associated with reducing macrophage invasion [9].

Accumulating evidence suggests that 4‐MU may be a valuable adjunctive therapy for treating ischemic stroke and other serious diseases [10–12]. Its multifaceted mechanism of action, encompassing anti‐inflammatory, antioxidant, and antiapoptotic properties, may provide a therapeutic advantage over single‐target agents [13]. Ultimately, the development of 4‐MU as a therapeutic agent for ischemic stroke may contribute to improved patient outcomes and a reduction in the socioeconomic burden of this devastating disease. Here, we tested whether 4‐MU can reduce cerebral I/R injury by modulating the activities of HAS1 and HAS2 in a rat model.

## 2. Materials and Methods

### 2.1. Animals and Experimental Design

Male Wistar rats (275 ± 15 g) were purchased from the Animal Center of Iran University of Medical Sciences (IUMS). Controlled conditions including a 12‐h light‐dark schedule at a temperature of 22^°^C ± 2^°^C, with unrestricted access to standard food and water, were selected for housing the animals. All rats underwent 3 days of handling. All protocols and procedures relevant to the animals were authorized by the Ethics Committee of IUMS (IR.IUMS.FMD.REC.1401.373) and were performed under the National Institutes of Health Guide for the Care and Use of Laboratory Animals.

Then, 116 rats were randomly assigned into four groups (*n* = 29 per group): sham, I/R, vehicle, and 4‐MU (25 mg/kg). In the sham group, animals were subjected only to surgical procedures without MCAO. In the I/R group, animals were subjected to MCAO for 30 min, and then, reperfusion was allowed for 24 h. In the vehicle group, animals were subjected to MCAO for 30 min, and at the onset of reperfusion, solvent (0.9% DMSO) was administered intraperitoneally. In the 4‐MU group, animals were subjected to MCAO for 30 min, and at the onset of reperfusion, 4‐MU (25 mg/kg) dissolved in 0.9% DMSO was administered intraperitoneally.

Only male Wistar rats were used to reduce variability from estrous cycle effects on ischemic outcomes, following established stroke research protocols. Future studies should include both sexes to address potential sex‐specific differences in 4‐MU efficacy.

The 25 mg/kg dose and timing at reperfusion onset were selected based on previous studies demonstrating optimal HAS inhibition at this concentration [8–12] and the therapeutic window for postischemic intervention. This dose has been shown to effectively reduce HA synthesis and provide neuroprotection in experimental stroke models without significant adverse effects.

### 2.2. Ischemic Stroke Model (MCAO)

The MCAO/R model was used to induce ischemia as described by Longa et al. [18]. Animals were anesthetized using a combination of ketamine (80 mg/kg) and xylazine (10 mg/kg) intraperitoneally. After isolation of the right common carotid artery, external carotid artery (ECA), and internal carotid artery (ICA), the distal end of the ECA was cut, and a 4‐0 silicon rubber‐coated monofilament with a tapered end was inserted into the right ICA and advanced approximately 18 ± 2 mm until slight resistance was felt, indicating blockage of the MCA. To permit reperfusion, the silicon rubber‐coated monofilament was removed after 30 min. Similar surgical procedures were performed for sham animals except for inserting the silicon rubber‐coated monofilament.

Animals were maintained at normothermia (37^°^C ± 0.5^°^C) throughout surgery using a feedback‐controlled heating pad. Anesthesia depth was monitored by toe pinch reflex. Postoperative analgesia was provided with buprenorphine (0.05 mg/kg, s.c.) every 12 h for 48 h. Animals were monitored for neurological deficits and weight loss daily. Cerebral blood flow monitoring was not performed, which represents a limitation of this study.

### 2.3. Automated Shuttle Box Test

Then, 7 days after surgery, rats underwent a shuttle box test. A shuttle box including a two‐compartment plexiglass box with a bright section and a dark section, with equal dimensions of the two sections (20 × 20 × 40 cm), was employed to assess learning and memory function. The instrument included stainless steel bars on the floor of both sections with a 1‐cm distance, a 100‐W light bulb 40 cm above the device′s bright side, and a guillotine door between the two sections.

The experiment was carried out in three steps:

1. Adaptation: Each rat was habituated to the apparatus for at least 5 min on two consecutive days prior to the start of the test.
2. Acquisition: On the third day, each rat was placed in the bright section, and the chamber was kept dark for 2 min. Both dark and bright sections were separated by a guillotine door. At the end of the period and after turning on the chamber′s light and opening the guillotine door, the time it took for the animal to enter from the light to the dark chamber was recorded by a chronometer and described as “initial latency.” Then, after closing the door, a single electric shock (1 mA for 1 s) was applied to the rat. Then, 1 min after the end of the experiment, the rat was transferred back to its cage. At this step, animals with a delay of more than 60 s were excluded from the experiment.
3. Retention and recall: This step was performed 24 h after the acquisition step on the fourth day. This step was similar to the prior step, but no shock was given when the rat entered the dark section. Step‐through latency (STL) was recorded at this point; STL is an indicator of how long the animal stayed in the light section before entering the dark section. The cut‐off time was 480 s if the animal did not move to the dark section.

### 2.4. Morris Water Maze (MWM) Test

Then, 7 days after surgery, rats underwent a MWM test. Spatial learning and memory performance were investigated using the MWM. The maze employed in this study consisted of a black circular pool (diameter = 150 cm and height = 60 cm) filled with water at 20^°^C ± 1^°^C to a height of 25 cm. The four quadrants of the black circular pool were arbitrarily described as southeast, southwest, northeast, and northwest. In the target quadrant (southeast), a hidden circular platform (diameter = 10 cm and located 1.5 cm below the water surface) was placed. During all days of the test, visual cues were placed around the maze, and recording of the swimming paths of rats during trials was performed using a camera fixed above the center of the maze.

Before each daily test, rats were placed in a dark test room for 30 min. Four trials each day for three consecutive days were performed for each animal. Each animal was subjected to a 20‐s habituation time on the platform on the first day only. Rats were placed in water facing the maze wall and allowed to swim for 90 s until they discovered the platform at the initiation of all trials. Rats that lacked the ability to find the platform were manually guided to it. After reaching the platform, they were permitted to remain for 20 s on the platform and then returned to their cage. A random selection for starting positions was made each day.

The probe (retention test) and visible platform test were performed on Day 4. To carry out the probe test, the escape platform was removed from the maze; afterward, rats were placed in water from a point opposite the target quadrant and allowed to swim for up to 60 s. Sensorimotor abilities of animals were measured using a visible platform test after the probe trial. Processing and analysis of the recorded behaviors of each rat were performed using a computerized system (Noldus EthoVision, Version 11.1).

Daily training sessions recorded escape latencies to assess learning acquisition over the 3‐day period. A visible platform test was conducted to evaluate sensorimotor function by measuring escape latency to a clearly visible platform. Representative swimming trajectories were recorded using EthoVision software to illustrate search strategies during both training and probe trials.

### 2.5. TTC Staining

Brain infarct volume was measured using TTC staining. After sacrificing the animals at the end of the experiment, brain tissues were separated and cut into 2‐mm coronal segments. To perform staining, brain sections were immersed in 2% TTC for 15 min at 37°C and fixed with 4% paraformaldehyde to increase contrast before taking photographs. Calculation of the percentage of infarct volume was performed based on the following formula:

Brain infarct volume %=left hemisphere volume−right hemisphere volume−infarct volume/left hemisphere volume×100%.

### 2.6. Western Blotting

Rat brain tissue proteins were extracted from ipsilateral peri‐infarct cortical regions using RIPA lysis buffer. A BCA protein assay kit was employed to measure protein concentrations. After separation of 50 *μ*g protein from each sample based on molecular weight by electrophoresis, proteins were transferred to PVDF membranes. After incubating the membranes with primary antibodies against HAS1 and HAS2 (1:1000 dilution) overnight at 4°C, they were incubated with corresponding HRP‐conjugated secondary antibodies for 2 h at 37°C. Color development of the PVDF membranes was performed using chemiluminescent HRP Substrate (Millipore). Quantification of protein values was performed using ImageJ software. *β*‐Actin was employed as the loading control.

### 2.7. Histopathological Examinations

Histopathological alterations of cerebral tissues were determined using Nissl staining. Brain tissues were fixed with 4% paraformaldehyde, embedded in paraffin, and cut into 5‐*μ*m slides before being Nissl stained and sealed. An optical microscope (Motic China Group Co., Ltd., BA210Digital, Xiamen, China) was used to investigate the pathological alterations in the CA1 region of the hippocampus.

Viable neurons in the CA1 region were quantified using systematic sampling (five fields per section and three sections per animal) at 400× magnification and expressed as neurons per 1000 *μ*m^2^.

### 2.8. Statistical Analysis

Findings were expressed as mean ± standard error of the mean (SEM). Analysis of all data was performed using one‐way ANOVA followed by Bonferroni′s test as a post hoc analytical test (GraphPad Prism 9.0 software). A *p* value less than 0.05 indicated a statistically significant difference.

## 3. Results

### 3.1. Infarct Volume

**Figure 1:**
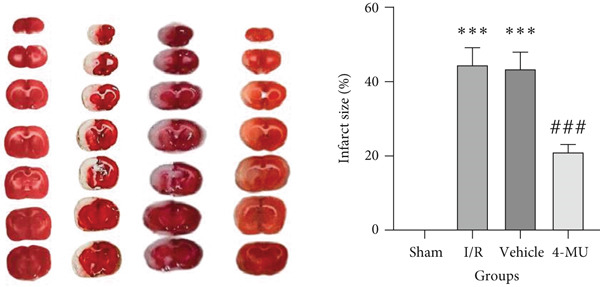
Representative images of TTC staining in different groups. Red color shows healthy regions, while white color demonstrates infarcted regions (*n* = 5 per group).  ^∗∗∗^ *p* < 0.001 vs. sham; ^###^ *p* < 0.001 vs. I/R and vehicle groups). Data were expressed as mean ± SEM.

Obvious cerebral infarction was evident in the I/R cohort compared to the sham group, indicating the successful establishment of the stroke model. As expected, a significant reduction in infarct size was observed in MCAO rats treated with 4‐MU (25 mg/kg) (Figure 1).

### 3.2. Passive Avoidance Test Performance

Significant reductions in STL were observed in animals exposed to MCAO compared to sham. 4‐MU (25 mg/kg) contributed to the restoration of STL compared to the I/R and vehicle groups. Moreover, MCAO rats exhibited significantly more time spent in the dark chamber than the sham. Treatment with only 0.9% DMSO did not change the time spent in the dark chamber, whereas treatment with 4‐MU (25 mg/kg) markedly decreased the time spent in the dark chamber compared to the I/R and vehicle cohorts (Figure 2a,b).

Figure 2Impact of 4‐MU on passive avoidance test performance. (a) STL and (b) time in dark chamber (*n* = 7).  ^∗∗∗^ *p* < 0.001,  ^∗∗^ *p* < 0.01, and  ^∗^ *p* < 0.05 vs. sham; ^###^ *p* < 0.001 vs. I/R and vehicle groups). Data were expressed as mean ± SEM.

**Figure:**
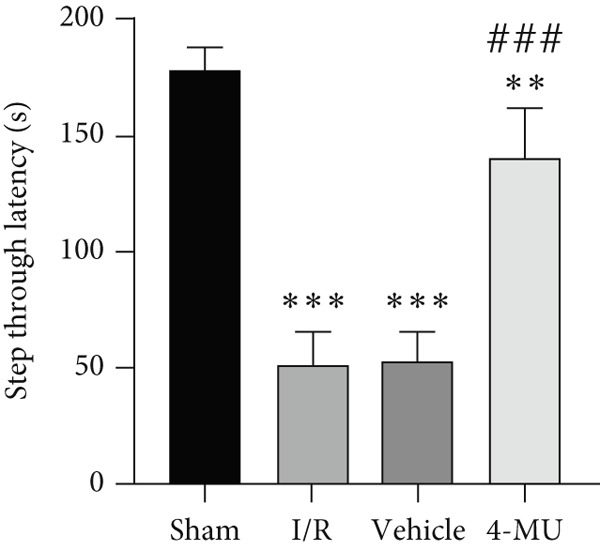
(a)

**Figure:**
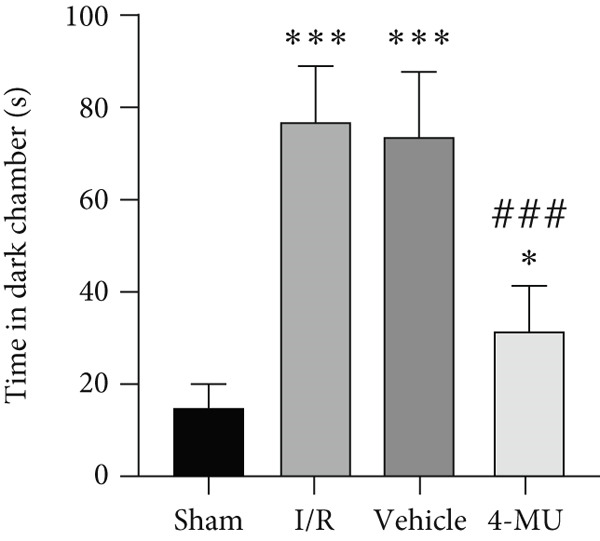
(b)

### 3.3. MWM Test Performance

**Figure 4:**
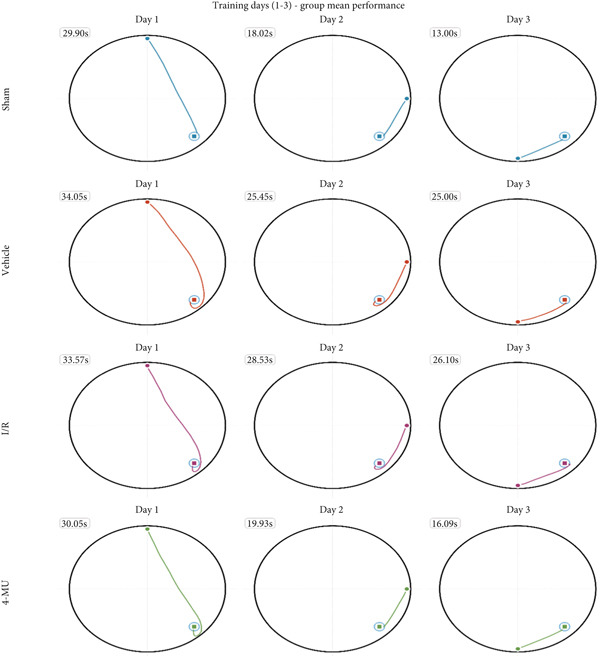
Representative swimming trajectories during Morris water maze training across Days 1–3. Group mean swim paths with escape latencies labeled. Platform location indicated by gray circle in southeast quadrant. Sham and 4‐MU groups show increasingly direct paths, while I/R and vehicle groups maintain circuitous patterns. *n* = 7 per group.

**Figure 5:**
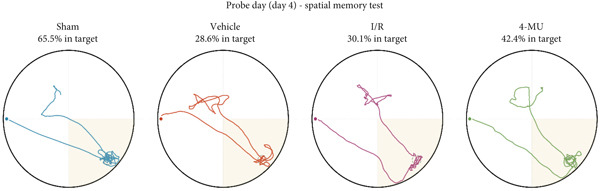
Probe trial swimming trajectories showing spatial memory performance. Individual swim paths during the 60‐s probe trial with the platform removed. Platform location indicated by a gray circle in the southeast quadrant. Percentage of time in the target quadrant shown above each trajectory (sham: 65.9%; I/R: 30.1%; vehicle: 28.6%; and 4‐MU: 42.4%). Scale bar = 0.2 m. *n* = 7 per group.

Reduced time spent in the target quadrant and increased latency time were observed in the I/R and vehicle cohorts compared to sham (Figure 3a). Treatment of I/R rats with 4‐MU (25 mg/kg) significantly improved the time spent in the target quadrant compared to the I/R and Vehicle cohorts. Training curve analysis demonstrated progressive learning acquisition over 3 days in all groups (Figure 3b). Sham animals showed rapid learning with decreasing escape latencies (Day 1: 29.9 ± 2.1 s → Day 2: 18.0 ± 1.6 s → Day 3: 13.0 ± 1.7 s, representing 56% improvement), while I/R (Day 1: 33.7 ± 2.6 s → Day 3: 26.0 ± 3.0 s, 23% improvement) and vehicle (Day 1: 34.1 ± 3.3 s → Day 3: 25.0 ± 2.4 s, 27% improvement) groups exhibited significantly impaired learning rates. 4‐MU treatment partially restored learning capacity (Day 1: 30.9 ± 3.5 s → Day 3: 16.0 ± 2.0 s, 48% improvement), demonstrating intermediate performance between sham and impaired groups. Representative swimming trajectories during training days illustrated the progressive development of spatial navigation strategies (Figure 4). Sham animals developed increasingly direct and efficient paths to the platform location across training days, while I/R and vehicle groups maintained circuitous, less efficient search patterns throughout the training period. 4‐MU‐treated animals demonstrated intermediate trajectory patterns, suggesting partial restoration of spatial learning ability. Swimming speed analysis revealed no significant differences among groups (Figure 3c: sham 27.5 ± 1.5 cm/s, I/R 25.5 ± 1.8 cm/s, vehicle 27.0 ± 1.5 cm/s, and 4‐MU 26.5 ± 1.3 cm/s; *p* > 0.05), confirming that cognitive differences were not attributable to motor impairments. Additionally, visible platform testing confirmed no sensorimotor differences between groups (escape latencies: sham 8.0 ± 2.0 s, I/R 9.1 ± 2.8 s, vehicle 9.0 ± 2.5 s, and 4‐MU 8.0 ± 2.1 s, *p* > 0.05), validating that spatial learning differences were not due to visual or motor deficits. Probe trial trajectory analysis revealed distinct spatial memory performance patterns among groups (Figure 5). Sham animals spent 65.9% of time in the target quadrant, demonstrating robust spatial memory with focused searching behavior in the platform location. In contrast, I/R (30.1%) and vehicle (28.6%) groups showed significantly impaired spatial memory characterized by dispersed exploration patterns across all quadrants. 4‐MU treatment improved target quadrant preference to 42.4%, indicating partial preservation of spatial memory with more focused search strategies compared to untreated ischemic groups.

Figure 3Impact of 4‐MU on behavioral parameters in the Morris water maze (MWM) test. (a) Time spent in the target quadrant during probe trial. (b) Escape latency across 3 training days. (c) Swimming speed. Visible platform testing confirmed no sensorimotor differences (Sham 8.0 ± 2.0 s, I/R 9.1 ± 2.8 s, vehicle 9.0 ± 2.5 s, and 4‐MU 8.0 ± 2.1 s; *p* > 0.05). *n* = 7 per group.  ^∗∗∗^ *p* < 0.001^,^^∗∗^ *p* < 0.01, and  ^∗^ *p* < 0.05 vs. sham; ^##^ *p* < 0.01 and ^#^ *p* < 0.05 vs. I/R and vehicle cohorts. Data were expressed as mean ± SEM.

**Figure:**
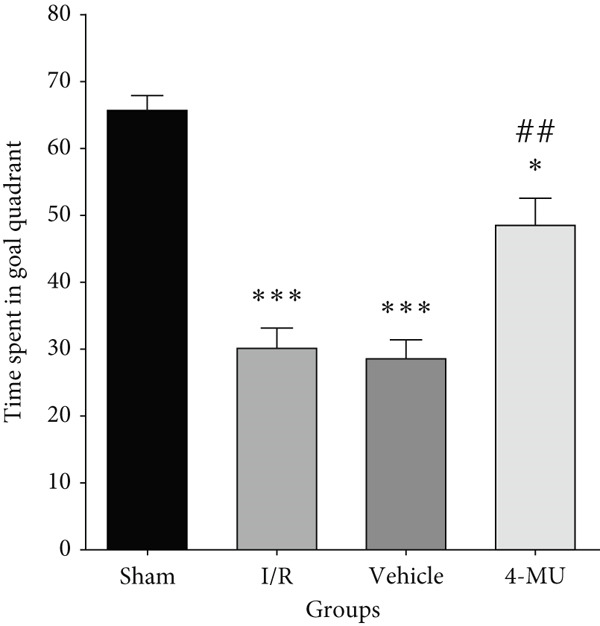
(a)

**Figure:**
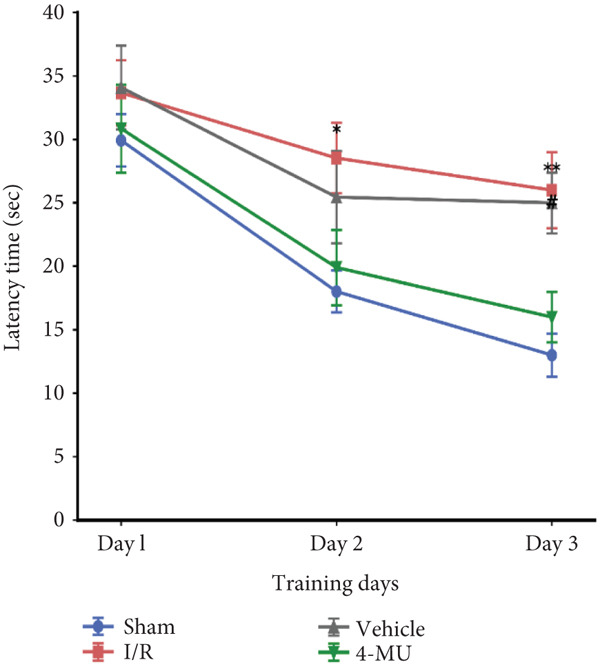
(b)

**Figure:**
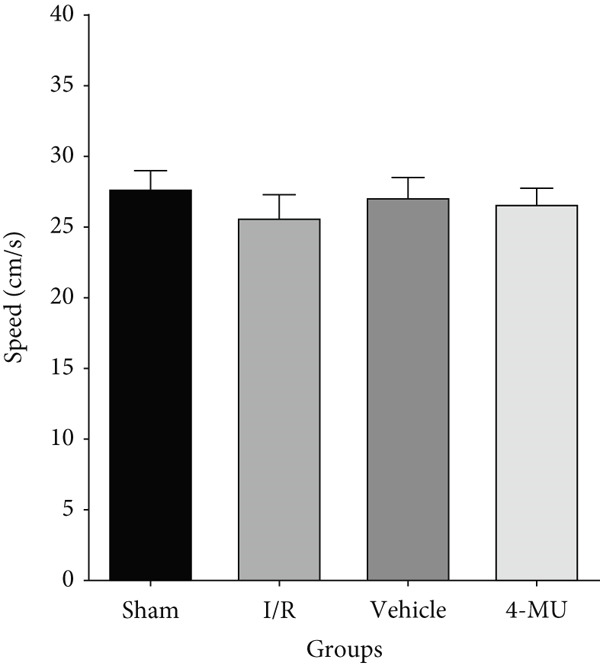
(c)

### 3.4. Expression of HAS1 and HAS2

HAS1 and HAS2 as targets of 4‐MU were found to be upregulated following MCAO compared to sham. Animals treated with 4‐MU (25 mg/kg) exhibited decreased expression levels of both HAS1 and HAS2 compared with the I/R and Vehicle cohorts (Figure 6a,b).

Figure 6Western blot analysis of HAS1 and HAS2 expression in brain tissue following cerebral ischemia–reperfusion injury. (a) HAS1 expression. (b) HAS2 expression. Protein samples extracted from ipsilateral peri‐infarct cortical regions. *β*‐Actin served as loading control. *n* = 5 per group.  ^∗∗∗^ *p* < 0.001 vs. sham; ^##^ *p* < 0.01 and ^#^ *p* < 0.05 vs. I/R and vehicle groups. Data were expressed as mean ± SEM.

**Figure:**
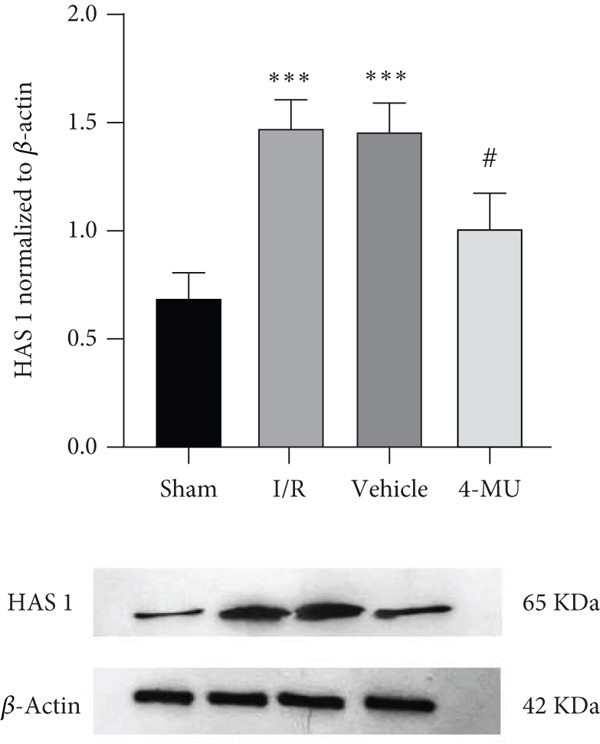
(a)

**Figure:**
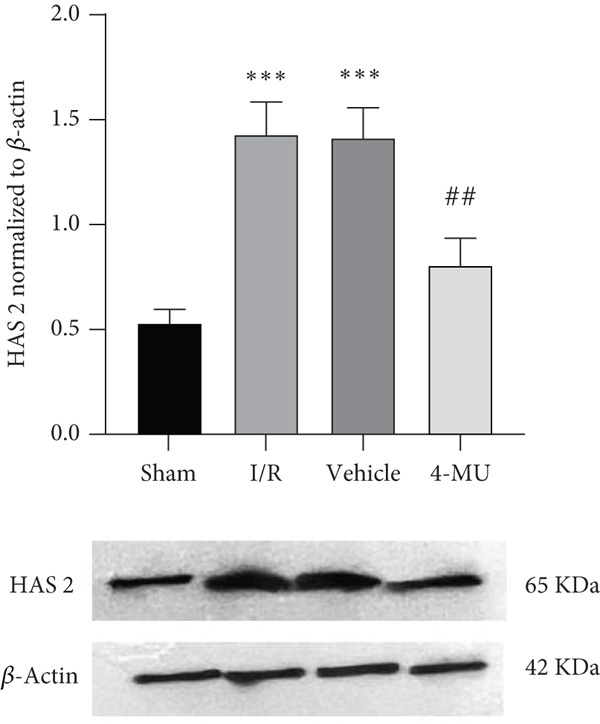
(b)

### 3.5. Neuronal Viability

**Figure 7:**
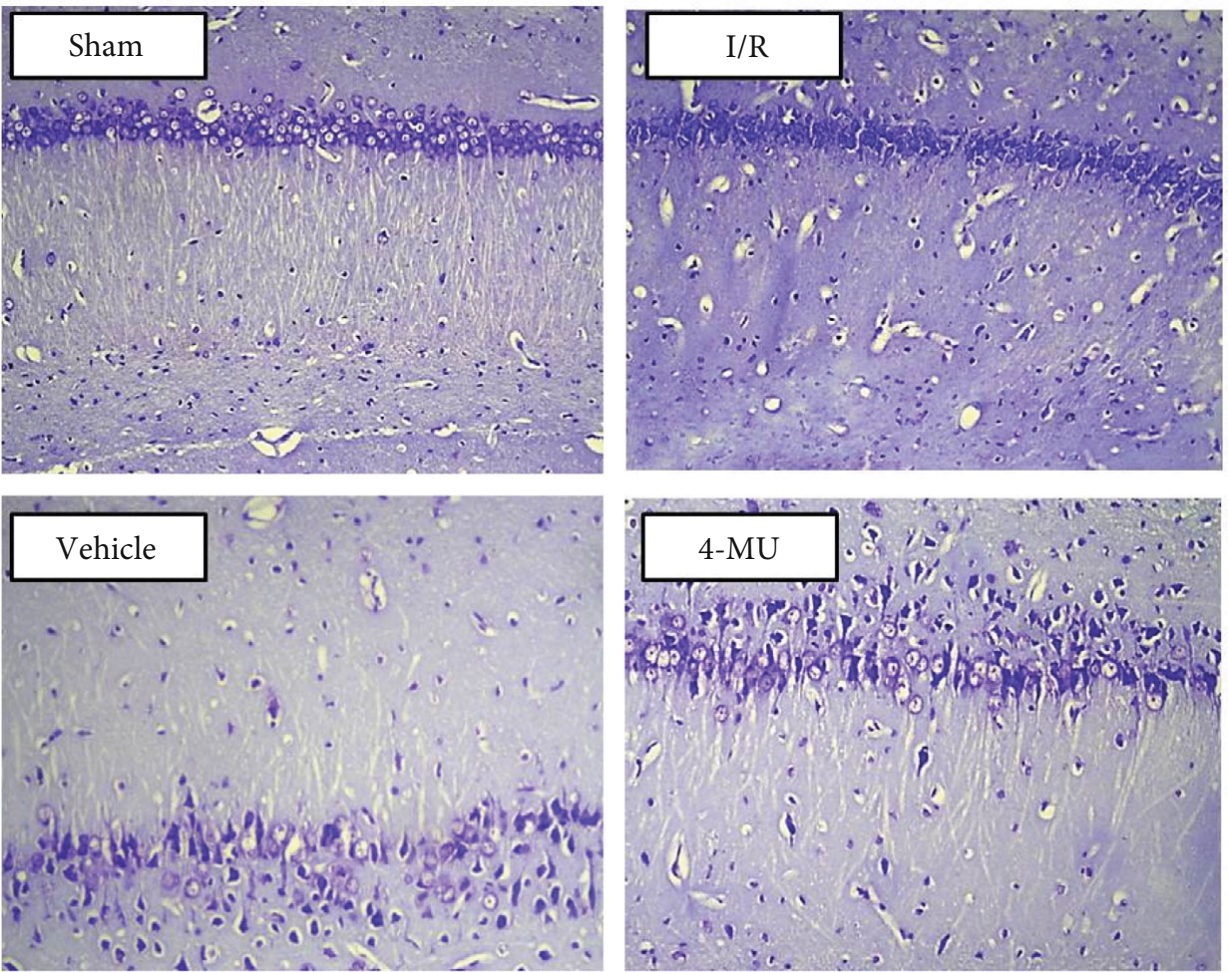
Representative images of Nissl staining in the CA1 region of hippocampus. Sham group shows intact neuronal cells with uniform staining. I/R and vehicle groups show scattered arrangement and nuclear deformation. 4‐MU treatment prevented cellular damage. Scale bar = 50 * μ*m. *n* = 5 per group.

Nissl staining of the CA1 region of the hippocampus revealed intact and tightly arranged neuronal cells with clear cytoplasm and nucleoli, exhibiting a uniform staining pattern in Sham animals (Figure 7). In contrast, scattered arrangement and nuclear deformation were observed in the I/R and vehicle cohorts compared to sham. Treatment with 4‐MU (25 mg/kg) significantly prevented cellular damage, with preserved neuronal morphology and organization.

**Figure 8:**
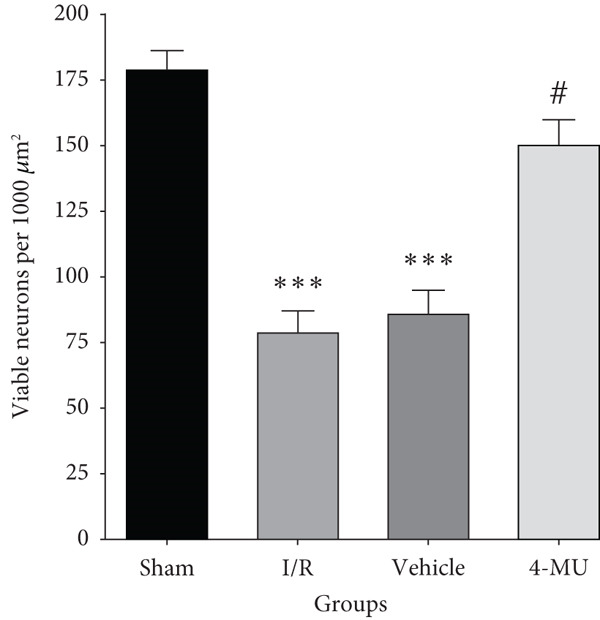
Quantitative analysis of viable neurons in the CA1 region of the hippocampus. Viable neurons per 1000 *μ*m^2^ showing significant neuronal loss following I/R injury with partial preservation by 4‐MU treatment. Quantification by systematic sampling (five fields per section, three sections per animal) at 400× magnification. *n* = 5 per group.  ^∗∗∗^ *p* < 0.001 vs. sham; ^##^ *p* < 0.01 and ^#^ *p* < 0.05 vs. I/R and vehicle groups. Data were expressed as mean ± SEM.

Quantitative analysis of Nissl‐stained sections revealed significant neuronal loss in the CA1 region following I/R injury (79 ± 8 viable neurons per 1000 *μ*m^2^) compared to sham controls (178 ± 8 neurons per 1000 *μ*m^2^, *p* < 0.001) (Figure 8). Treatment with 4‐MU partially preserved neuronal viability (150 ± 10 neurons per 1000 *μ*m^2^, *p* < 0.05 vs. I/R group), demonstrating neuroprotective effects consistent with the behavioral improvements observed. Vehicle treatment showed no significant difference from the I/R group (86 ± 9 neurons per 1000 *μ*m^2^), confirming that the protective effects were specific to 4‐MU rather than the solvent.

## 4. Discussion

In addition to physical disabilities, cerebral I/R injury causes learning and memory impairments that heavily influence functional recovery poststroke [7, 14]. In the present study, our findings indicated that treatment with 4‐MU (25 mg/kg) significantly attenuated infarct volume and reductions in STL, along with elevated time in the dark chamber, indicating learning and memory improvement. Consistent with our results, Dubisova et al. reported that oral administration of 4‐MU attenuated perineuronal nets and improved recognition memory in mice [7].

Moreover, we found that the beneficial effects of 4‐MU in addressing I/R injury are associated with the inhibition of both HAS1 and HAS2. Emerging evidence demonstrates that 4‐MU can reduce damage following I/R by suppressing oxidative stress and downregulating TLR4/NF‐*κ*B/NLRP3 [15]. Hyaluronic acid is a major ingredient of the brain extracellular matrix and a modulator of cell differentiation, migration, and angiogenesis. Prior investigations have found that HA accumulation in stroke‐affected regions is linked to the overexpression of Hyaluronidase‐1 and Hyaluronidase‐2 from 1 h to 21 days after stroke [16].

A clinical study by Al′Qteishat et al. showed that the expression and concentrations of the enzymes responsible for HA synthesis and degradation markedly increased in post‐mortem tissue and serum of patients at 1, 3, 7, and 14 days following stroke [7]. Under homeostatic conditions, low HA‐binding capacity has been reported for most immune cells. In inflammatory conditions, elevated HA binding of activated immune cells occurs via CD44 [15, 17]. Increased expression of HAS isoenzymes (HAS1–3) during inflammation contributes to a predominance of low‐molecular‐weight HA, thereby activating macrophages through TLRs, which in turn results in elevated inflammation at the site of injury [16].

These previous investigations are compatible with our data indicating that tissue damage from MCAO resulted in upregulation of HAS1 and HAS2, subsequently leading to obvious infarct volume and cell death in the CA1 region of the hippocampus. Such abnormalities were reversed by treatment with 4‐MU (25 mg/kg), suggesting protective effects against cerebral I/R injury through inhibition of HAS1 and HAS2.

## 5. Conclusion

Ischemic stroke is a leading cause of morbidity and mortality worldwide and is characterized by the obstruction of blood flow to the brain, resulting in neuronal death and tissue damage. HAS enzymes are responsible for synthesizing HA, a glycosaminoglycan that plays a crucial role in regulating inflammation, cell migration, and tissue repair. 4‐MU is a naturally occurring compound shown to inhibit HAS activity, thereby reducing HA production. The purpose of this study was to investigate the current understanding of the role of HAS in ischemic stroke and discuss the potential therapeutic effects of 4‐MU on ischemic stroke through HAS inhibition.

The neuroprotective effects of 4‐MU in ischemic stroke are thought to be mediated through inhibiting HAS and subsequently reducing HA production. Additionally, 4‐MU has been shown to reduce infarct size, improve neurological function, and enhance cognitive recovery in animal models of MCAO. In this study, we found that 4‐MU may be a promising candidate for reducing cerebral I/R injury and improving learning and memory impairments in a rat model of ischemic stroke. It seems likely that the neuroprotective impact of 4‐MU in addressing cerebral I/R injury is related to suppressing the activities of HAS1 and HAS2.

**Nomenclature**
4‐MU: 4‐methylumbelliferone
MCAO: middle cerebral artery occlusion
HAS: hyaluronan synthase
I/R: ischemia–reperfusion

## Ethics Statement

All animal procedures were approved by the Ethics Committee of Iran University of Medical Sciences (IR.IUMS.FMD.REC.1401.373) and conducted in accordance with the National Institutes of Health Guide for the Care and Use of Laboratory Animals.

## Disclosure

A preprint has previously been published [18].

## Conflicts of Interest

The authors declare no conflicts of interest.

## Funding

This study was supported by the Iran University of Medical Sciences, 10.13039/100012021, 1401.378.

## Data Availability

The data that support the findings of this study are available from the corresponding author upon reasonable request.

## References

[1] Jokinen H. , Melkas S. , Ylikoski R. , Pohjasvaara T. , Kaste M. , Erkinjuntti T. , and Hietanen M. , Post-Stroke Cognitive Impairment Is Common Even After Successful Clinical Recovery, European Journal of Neurology. (2015) 22, no. 9, 1288–1294, 10.1111/ene.12743, 2-s2.0-84939471282, 26040251. 26040251

[2] Bacigaluppi M. , Comi G. , and Hermann D. M. , Animal Models of Ischemic Stroke. Part Two: Modeling Cerebral Ischemia, Open Neurology Journal. (2010) 4, no. 1, 34–38, 10.2174/1874205X01004010034. 20721320 PMC2923341

[3] Tamouk S. , Jahangiri H. M. , Jahromi E. K. , Hamblin M. R. , Ramezani F. , and Aboutaleb N. , 4-Methyllumbiferone (4-MU) Exerts a Neuroprotective Effect Against Cerebral Ischemia/Reperfusion Injury by Ameliorating Learning and Memory Impairments, 2024, bioRxiv.

[4] Van Der Flier W. M. , Skoog I. , Schneider J. A. , Pantoni L. , Mok V. , Chen C. L. H. , and Scheltens P. , Vascular Cognitive Impairment, Nature Reviews Disease Primers. (2018) 4, no. 1, 10.1038/nrdp.2018.3, 2-s2.0-85042226113. 29446769

[5] Pantoni L. , Have Stroke Neurologists Entered the Arena of Stroke-Related Cognitive Dysfunctions? Not Yet, but They Should!, Stroke. (2017) 48, no. 6, 1441–1442, 10.1161/STROKEAHA.117.016869, 2-s2.0-85019589905. 28487330

[6] Kalaria R. N. , Akinyemi R. , and Ihara M. , Stroke Injury, Cognitive Impairment and Vascular Dementia, Biochimica et Biophysica Acta (BBA)-Molecular Basis of Disease. (2016) 1862, no. 5, 915–925, 10.1016/j.bbadis.2016.01.015, 2-s2.0-84961725735. 26806700 PMC4827373

[7] Al’Qteishat A. , Gaffney J. , Krupinski J. , Rubio F. , West D. , Kumar S. , Kumar P. , Mitsios N. , and Slevin M. , Changes in Hyaluronan Production and Metabolism Following Ischaemic Stroke in Man, Brain. (2006) 129, no. 8, 2158–2176, 10.1093/brain/awl139, 2-s2.0-33745880779, 16731541. 16731541

[8] Katarzyna Greda A. and Nowicka D. , Hyaluronidase Inhibition Accelerates Functional Recovery From Stroke in the Mouse Brain, Journal of Neurochemistry. (2021) 157, no. 3, 781–801, 10.1111/jnc.15279, 33345310. 33345310

[9] Nagy N. , Kaber G. , Haddock N. L. , Hargil A. , Rajadas J. , Malhotra S. V. , Unger M. A. , Frymoyer A. R. , and Bollyky P. L. , The Pharmacokinetics and Pharmacodynamics of 4-Methylumbelliferone and Its Glucuronide Metabolite in Mice, Hyaluronan: Structure, Biology and Biotechnology, 2023, Springer, 161–188, 10.1007/978-3-031-30300-5_8.

[10] Onufriev M. V. , Moiseeva Y. V. , Zhanina M. Y. , Lazareva N. A. , and Gulyaeva N. V. , A Comparative Study of Koizumi and Longa Methods of Intraluminal Filament Middle Cerebral Artery Occlusion in Rats: Early Corticosterone and Inflammatory Response in the Hippocampus and Frontal Cortex, International Journal of Molecular Sciences. (2021) 22, no. 24, 13544, 10.3390/ijms222413544, 34948340. 34948340 PMC8703333

[11] Gao Y. , Ya B. , Li X. , Guo Y. , and Yin H. , Myricitrin Ameliorates Cognitive Deficits in MCAO Cerebral Stroke Rats Via Histone Acetylation-Induced Alterations of Brain-Derived Neurotrophic Factor, Molecular and Cellular Biochemistry. (2021) 476, no. 2, 609–617, 10.1007/s11010-020-03930-4, 33074446. 33074446

[12] Zhan Y. , Li M. Z. , Yang L. , Feng X. F. , Lei J. F. , Zhang N. , Zhao Y. Y. , and Zhao H. , The Three-Phase Enriched Environment Paradigm Promotes Neurovascular Restorative and Prevents Learning Impairment After Ischemic Stroke in Rats, Neurobiology of Disease. (2020) 146, 105091, 10.1016/j.nbd.2020.105091, 32979506. 32979506

[13] Dubisova J. , Burianova J. S. , Svobodova L. , Makovicky P. , Martinez-Varea N. , Cimpean A. , Fawcett J. W. , Kwok J. C. F. , and Kubinova S. , Oral Treatment of 4-Methylumbelliferone Reduced Perineuronal Nets and Improved Recognition Memory in Mice, Brain Research Bulletin. (2022) 181, 144–156, 10.1016/j.brainresbull.2022.01.011, 35066096. 35066096 PMC8867078

[14] Al Qteishat A. , Gaffney J. J. , Krupinski J. , and Slevin M. , Hyaluronan Expression Following Middle Cerebral Artery Occlusion in the Rat, Neuroreport. (2006) 17, no. 11, 1111–1114, 10.1097/01.wnr.0000227986.69680.20, 2-s2.0-33745882359, 16837837. 16837837

[15] Kuipers H. F. , Rieck M. , Gurevich I. , Nagy N. , Butte M. J. , Negrin R. S. , Wight T. N. , Steinman L. , and Bollyky P. L. , Hyaluronan Synthesis Is Necessary for Autoreactive T-Cell Trafficking, Activation, and Th1 Polarization, Proceedings of the National Academy of Sciences. (2016) 113, no. 5, 1339–1344, 10.1073/pnas.1525086113, 2-s2.0-84956677725, 26787861. PMC4747722 26787861

[16] Grandoch M. , Bollyky P. L. , and Fischer J. W. , Hyaluronan: A Master Switch Between Vascular Homeostasis and Inflammation, Circulation Research. (2018) 122, no. 10, 1341–1343, 10.1161/CIRCRESAHA.118.312522, 2-s2.0-85052945171. 29748364

[17] Hathcock K. S. , Hirano H. , Murakami S. , and Hodes R. J. , CD44 Expression on Activated B Cells. Differential Capacity for CD44-Dependent Binding to Hyaluronic Acid, Journal of Immunology. (1993) 151, no. 12, 6712–6722, 10.4049/jimmunol.151.12.6712, 7505013. 7505013

[18] Tamouk S. , Jahangiri H. M. , Jahromi E. K. , Hamblin M. R. , Ramezani F. , and Aboutaleb N. , 4-Methyllumifrone (4-MU) Can Improve Learning and Memory After Cerebral Ischemia/Reperfusion Injury in Rats, 2024, bioRxiv, 10.1101/2024.11.29.626098. PMC12680788 41355840

